# Profiling Non-Coding RNA Changes Associated with 16 Different Engineered Nanomaterials in a Mouse Airway Exposure Model

**DOI:** 10.3390/cells10051085

**Published:** 2021-05-01

**Authors:** Joseph Ndika, Piia Karisola, Pia Kinaret, Marit Ilves, Harri Alenius

**Affiliations:** 1 Human Microbiome Research (HUMI), Faculty of Medicine, University of Helsinki, 00014 Helsinki, Finland; piia.karisola@helsinki.fi (P.K.); Marit_Ilves@hms.harvard.edu (M.I.); harri.alenius@helsinki.fi (H.A.); 2 Institute of Biotechnology, Helsinki Institute of Life Science, University of Helsinki, 00014 Helsinki, Finland; pia.kinaret@helsinki.fi; 3 Faculty of Medicine and Health Technology, Tampere University, 33100 Tampere, Finland; 4 Institute of Environmental Medicine, Karolinska Institutet, 17177 Stockholm, Sweden

**Keywords:** nanoparticles, toxicogenomics, long non-coding RNA

## Abstract

Perturbations in cellular molecular events and their associated biological processes provide opportunities for hazard assessment based on toxicogenomic profiling. Long non-coding RNAs (lncRNAs) are transcribed from DNA but are typically not translated into full-length proteins. Via epigenetic regulation, they play important roles in organismal response to environmental stress. The effects of nanoparticles on this important part of the epigenome are understudied. In this study, we investigated changes in lncRNA associated with hazardous inhalatory exposure of mice to 16 engineered nanomaterials (ENM)–4 ENM (copper oxide, multi-walled carbon nanotubes, spherical titanium dioxide, and rod-like titanium dioxide particles) with 4 different surface chemistries (pristine, COOH, NH_2_, and PEG). Mice were exposed to 10 µg of ENM by oropharyngeal aspiration for 4 consecutive days, followed by cytological analyses and transcriptomic characterization of whole lung tissues. The number of significantly altered non-coding RNA transcripts, suggestive of their degrees of toxicity, was different for each ENM type. Particle surface chemistry and shape also had varying effects on lncRNA expression. NH_2_ and PEG caused the strongest and weakest responses, respectively. Via correlational analyses to mRNA expression from the same samples, we could deduce that significantly altered lncRNAs are potential regulators of genes involved in mitotic cell division and DNA damage response. This study sheds more light on epigenetic mechanisms of ENM toxicity and also emphasizes the importance of the lncRNA superfamily as toxicogenomic markers of adverse ENM exposure.

## 1. Introduction

Due to their ongoing contributions to sustainable nanotechnology-based innovations, engineered nanomaterials (ENM) are still being produced in bulk quantities worldwide [1]. Workers and consumers continuously come in contact with a diverse array of potentially hazardous substances. Biological toxicity of ENM depends on the size and shape of the core material and also on the used surface chemistry. Carboxylation (-COOH) and especially polyethylene glycosylation (PEG) are often shown to reduce bioactivity and pathogenicity of ENM, while amination (-NR_3_^+^) is sometimes reported to even enhance toxicity of the same particles when compared to its pristine (unmodified or core) form [2,3,4,5]. Titanium dioxide nanoparticles (TiO_2_) are the most manufactured nanomaterials, while ion-releasing copper oxide (CuO) and multiwalled carbon nanotubes (MWCNT) have unique characteristics making them commercially exciting materials. We have recently shown that core material chemistry and surface modifications of these 16, and 12 other nanosized particles had varying immunomodulatory effects in the mouse airways [6]. Amination rendered the strongest inflammatory response, which was usually suppressed by PEGylation [6]. The results also demonstrated that, for each material, the magnitude of transcriptomic changes (number of differentially expressed genes) was well correlated to the extent of inflammatory cell infiltration into the lungs, irrespective of the material surface functional group [5,6].

Protein-coding genes make up only 2% of the genome in humans [7], and their numbers are not significantly higher than those in much simpler eukaryotes. However, high throughput sequencing has revealed that the human genome is extensively transcribed. This non-coding transcriptome as well as differential splicing, are now accepted as key contributors to the complexity of mammalian physiology [8]. Non-coding RNAs (ncRNAs) are classified according to their length and activity [9], with many classes revealed as essential regulators of gene expression through a variety of mechanisms [10]. Long non-coding RNAs (lncRNAs) are a class of ncRNA that are longer than 200 nucleotides with little to no protein coding potential. LncRNAs interact with other coding and non-coding RNAs, DNAs, and proteins [11]. Due to technological advances in next-generation sequencing, lncRNAs are increasingly being identified and more than 120,000 lncRNA transcripts have thus far been revealed in the human genome (www.LNCipedia.org, accessed 1 March 2021). Abnormal expression of lncRNA has been observed in response to environmental stress (pesticides, persistent environmental chemicals, UV radiation, and heavy metals) [12]. As such, lncRNA expression as a novel paradigm for epigenetic toxicology has been proposed [13].

For proper hazard and risk assessment of ENM, the toxicological mechanisms relating to different ENM physicochemical parameters and immunomodulatory potential, need to be elucidated. In vivo, gene expression profiling of mouse lung exposed to CuO, MWCNT, TiO_2_p (spherical shape), and TiO_2_r (rodlike shape) ENM with different surface chemistries (pristine, COOH, NH_2_ and PEG) reveal variable biological responses [5,6]. In this study, we use a multi-omic profiling strategy to reveal which, if any, of these adverse ENM-induced biological processes are triggered by lncRNA-dependent mechanisms, and whether there is material- and surface chemistry-specific differences in epigenetic expression of lncRNA.

## 2. Materials and Methods

### 2.1. Panel of Nanomaterials

All 16 engineered nanomaterials studied were provided by the FP7-NANOSOLUTIONS consortium. Their synthesis, functionalization, and characterization are extensively described elsewhere [6,14,15]. Following the NANOSOLUTIONS standard operating procedures provided for each material, endotoxin free water (HyClone, HyPure Cell Culture Grade Water, Thermo Scientific, Waltham, MA, USA) was used for all ENM dispersions in glass tubes. The presence of functional groups was confirmed with XPS [6]. The dilutions for animal exposures were prepared in sterile PBS (200 µL/mL), in ultra clean conditions, with sterile equipment. Control samples were prepared in pure PBS (CuO, TiO_2_r, and TiO_2_p) or PBS + 0.1% BSA (MWCNT) depending on how its corresponding ENM was dispersed.

### 2.2. Study Design and Sampling

Detailed description of the animal exposures and extraction of lung tissue RNA are provided elsewhere [6]. Mouse models were used because they provide more realistic air–liquid interface exposure to particles, as opposed to using submerged cell cultures. In addition, more toxicological endpoints such as immune cell infiltration and histological/cytological evaluation of lung tissue can be assessed. Mice were exposed to each ENM at a dose of 10 µg per day for 4 consecutive days. This dose mimics work-place exposure to a deposited cumulative dose of 40 µg, which can be achieved in 1 week at permissible exposure limits of 5 mg/m^3^ defined by Occupational Safety & Health Administration. The calculations for this mouse–human dose equivalent extrapolation are derived from Yanamala and colleagues [16]. After exposures, total RNA was isolated and purified from mouse lung samples via the phenol/chloroform method. RNA samples with RNA integrity values >7.5 were diluted in ultrapure sterile water to 200 ng in 1.5 μL. Two-color microarray-based gene expression analysis (Quick amp labelling kit, two-color, Agilent, Santa Clara, CA, USA) was performed using Agilent’s Sure Print G3 Mouse, GE8 × 60K DNA microarrays. Hybridized slides were scanned (DNA microarray scanner, model G2505C, Agilent), and the raw data were extracted using Agilent’s feature extraction software (V12.0.1.1). The expression data are available in Gene Expression Omnibus with the accession number GSE157266.

### 2.3. Data Processing LncRNA Expression Analysis

SurePrint G3 Mouse Gene Expression Microarrays provide comprehensive coverage of genes and transcripts using the latest annotation databases. This array features complete coverage of established RefSeq coding transcripts and long non-coding RNA (lncRNA), thus ensuring in essence that two distinct layers of the transcriptome (protein coding genes and long non-coding RNA) can be investigated in a single microarray experiment. Changes in gene expression were analyzed with an R-based graphical user interface composed of standard bioinformatics packages-eUTOPIA [17]. As a first step, we performed log2 transformation of probe median foreground intensities. Because lncRNAs have an overall lower expression when compared to protein coding genes [18], in order to retain the majority of lncRNA transcripts for differential expression analysis, all probes with intensities exceeding background signal (negative control probes) in at least half of the samples were retained in the data frame. After quantile normalization, batch effects due to labeling and array-specific variance were removed using the ComBat method [19]. Accession numbers were used as annotation of choice for differential expression analysis, since the majority of lncRNAs do not have gene symbols. Between-group differential expression was performed by Limma Model analysis, using Benjamin & Hochberg method for multivariate correction of false discovery rate (FDR). A minimum log2 difference of 0.58, and an FDR of at most 5% was implemented as cut-off to consider a gene as significantly differentially expressed between exposed and control mice. Protein coding genes were filtered out from the list of differentially expressed genes using their RefSeq and/or ENSEMBL identifiers. Perseus graphical user interface [20] was used to generate clusters and heatmaps of differentially expressed lncRNAs. Clustering parameters used were as follows: Distance: Euclidean, Linkage: Average, and Cluster Preprocessing: K-means.

### 2.4. LncRNA Functional Prediction

Using differentially expressed mRNA genes from the same samples (fold change above 1.5 at a maximum FDR of 5%), lncRNAs that were highly correlated (Pearson’s correlation coefficient, *p* value < 0.05 and R > |0.8|) to 15 or more genes were identified. The cutoff of 15 genes was chosen to prioritize identification of potential networks of lncRNA-regulated genes. The physiological implications of these lncRNA-associated genes were inferred via biological process enrichment analyses using g:Profiler—a web server for functional enrichment analysis [21].

## 3. Results

### 3.1. LncRNA Expression Profiles Are Unique for Each Nanomaterial Type

**Figure 1:**
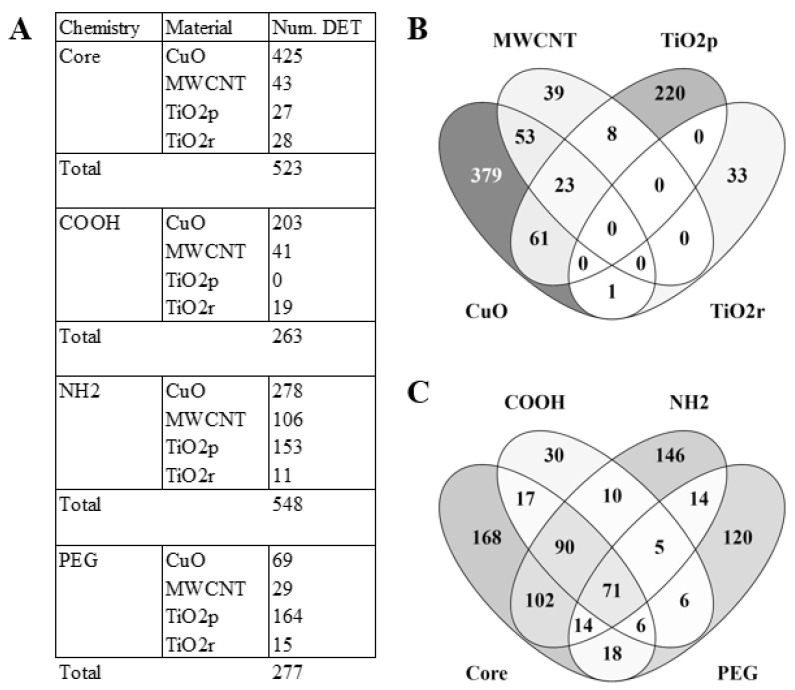
Comparison of most biologically relevant differentially expressed (DE) lncRNA transcripts across the different materials and surface chemistry types. In (**A**), the number of DE transcripts is shown for all engineered nanomaterials, across all the different material surface chemistry groups. Venn comparisons reveal both unique and shared lncRNA expression signatures between ENM (**B**) and ENM surface chemistries (**C**).

To answer whether ENM exposure triggers material-specific changes in lncRNA expression, total RNA from lung tissue of ENM-exposed mice were each compared to controls. A total of 4222 lncRNA transcripts were identified as significantly (Benjamini-Hochberg q-value < 0.05) differential expressed (DE). Principal component analyses, based on these DE transcripts, separated all ENM-exposed samples from control samples (Figure S1). The median change in expression of all significantly DE lncRNA transcripts was 33% (i.e., log 2 difference of 0.41). As such, we implemented a 1.5-fold change (log 2 difference of 0.58) cut-off to prioritize DE lncRNA transcripts with potential biological relevance. This narrowed down the number of unique significantly DE lncRNA transcripts across all exposures to 817. LncRNA expression varied both according to the type of material the animal was exposed to, as well as its surface chemistry (Figure 1A). For example, CuO engineered nanomaterials accounted for the most DE lncRNA transcripts, followed by TiO_2_p, MWCNT, and then TiO_2_r. With respect to the effect of differential surface chemistries on lncRNA expression, COOH and PEG functional groups triggered only about half as many lncRNA transcripts as the NH_2_ and Core (unmodified) ENM surface chemistries (Figure 1A). Although only 18% of the DE lncRNAs transcripts are shared between the ENM exposures, greater similarity could be observed across the different ENM surface chemistries, where 44% of the DE lncRNAs transcripts overlap (Figure 1B–C).

**Figure 2:**
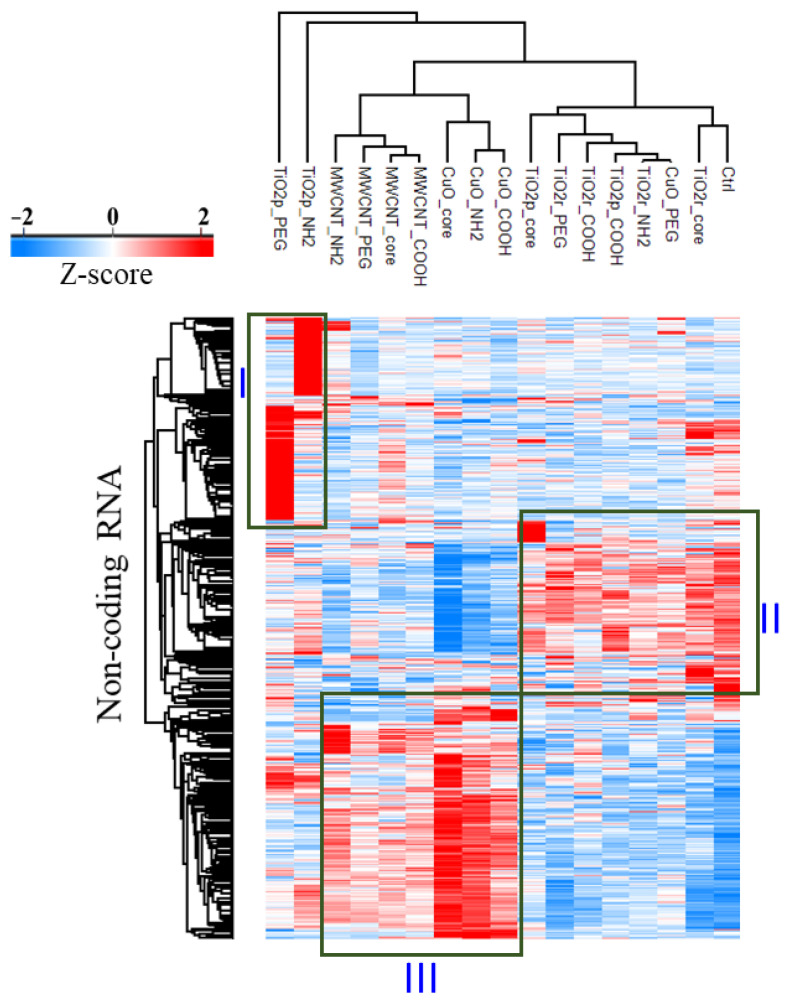
Heatmap of differentially expressed lncRNA transcripts (1.5-fold change, q-value 0.05). Hierarchical clustering reveals 3 distinct clusters of lncRNAs. Boxes highlight a group of lncRNA transcripts that are upregulated in each cluster (**I**–**III**). Each column represents the average Z-scored intensity value from 3 biological replicates.

A hierarchical cluster based on the relative expression of these 817 lncRNAs, reveals 3 separate lncRNA clusters (Figure 2). Cluster I consists of lncRNA transcripts that are predominantly upregulated in either TiO_2_p-NH_2_ or TiO_2_p-PEG. Transcripts that are most abundantly expressed in the negative control exposures, CuO-PEG, TiO_2_p-COOH, TiO_2_p-Core, and all TiO_2_r exposures are found in Cluster II. The third cluster consists of lncRNA transcripts that are upregulated in all MWCNT exposures, CuO-Core, CuO-COOH, and CuO-NH_2_ exposures.

### 3.2. The Magnitude of ENM-Induced Changes in LncRNA Expression Is Consistent with mRNA Expression from the Same Exposures

Because we used an array with probes for both lncRNA and mRNA we could co-evaluate changes in mRNA as well lncRNA from the same total RNA sample. We next asked whether the number of modulated lncRNA (exposure severity) is consistent with what was observed at the level of mRNA. In addition to the 817 DE lncRNAs identified, 3237 mRNA transcripts were identified as DE across all ENM exposures relative to controls. The highest number of DE protein-coding genes (mRNA) and lncRNAs were triggered by exposure to CuO ENM, and the lowest by TiO_2_r ENM. A scatter plot of the number of DE lncRNA against the number of DE mRNA showed good concordance (Spearman’s rank correlation, 0.96) between both layers of the transcriptome (Figure S2A). Additionally, in contrast to lncRNA, MWCNT were more toxic (number of DE genes) than TiO_2_p on the mRNA level (Figure S2B,C).

### 3.3. Integration of the Long Non-Coding and Protein-Coding Transcriptome Layers Identifies Potential Co-Regulated lncRNA/mRNA Networks

**Figure 3:**
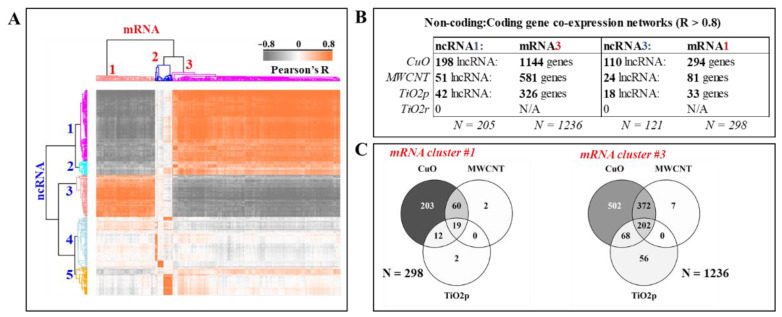
Co-regulated lncRNA-mRNA networks, triggered by hazardous copper oxide (CuO), multiwalled carbon nanotubes (MWCNT) and titanium dioxide (TiO_2_p/TiO_2_r) engineered nanomaterial exposures. Hierarchical clustering based on Pearson’s correlation coefficients between differentially expressed lncRNA and mRNA transcripts, reveals 5 clusters of lncRNA transcripts that are highly correlated (−0.8 < R > 0.8) to at least 15 genes (mRNA) (**A**). The positively correlated mRNA-lncRNA transcript pairs from the top two correlated clusters; lncRNA (cluster #1 & #3) and mRNA (cluster #1 & #3) are shown in (**B**). No differentially expressed lncRNA transcripts from the TiO2r exposures are correlated (−0.8 < R > 0.8) to differentially expressed genes. Distribution of lncRNA-associated genes between the CuO, MWCNT, and TiO_2_p exposures are shown in (**C**). Shared and unique lncRNA-associated differentially expressed genes indicate both common and unique epigenetic mechanisms of toxicity.

To answer whether DE lncRNA transcripts potentially trigger downstream changes in mRNA levels, a correlation analysis between these two layers of the transcriptome was performed. DE lncRNAs (817 transcripts) were assessed for association to DE mRNA (3237 genes), from the same set of exposure/control contrast sets. Only DE lncRNA with a strong association (abs Pearson’s R > 0.8) to at least 15 mRNA genes were retained in the data matrix. All mRNA transcripts with an abs correlation coefficient, R > 0.8, to at least 1 lncRNA transcript were also retained. The output from this correlation matrix is depicted as a heatmap and table in Figure 3A,B. A Venn distribution of the lncRNA-associated differentially expressed genes is shown in Figure 3C. The highest number of lncRNA transcript clusters with a strong correlation (−0.8 < R > 0.8) to at least 15 genes was identified in the transcriptome of mice lung exposed to CuO nanoparticles (198 + 110 lncRNA transcripts), followed by MWCNT (51 + 24 lncRNA transcripts) and TiO2p (42 + 18 transcripts). None of the DE lncRNA transcripts in TiO2r exposures were identified as strongly correlated to any mRNA gene. In total, the top two correlated clusters of mRNA-lncRNA transcript pairs accounted for 40% (lncRNA; 205 + 121 transcripts) and 47% (mRNA; 298 + 1236 transcripts) of all differentially expressed.

The top two clusters of mRNA-associated lncRNA transcripts (cluster #1–205 lncRNA transcripts and cluster #3–121 lncRNA transcripts) were opposingly correlated to the two separate clusters of mRNA transcripts; #1–298 genes and #2–1236 genes. This was explained by evaluation of the relative median expression intensities (Figure S3). While the relative expression of the lncRNA transcripts in cluster #1 is lowest in controls and highest in non-PEGylated MWCNT and CuO exposures (Figure S3, left panel), the reverse is true of the lncRNAs in cluster #3 (Figure S3, right panel).

### 3.4. The Genes Are Potentially Co-Regulated as mRNA-lncRNA Network Clusters and Are Predominantly Involved in ENM-Associated Cellular DNA Damage Response

**Figure 4:**
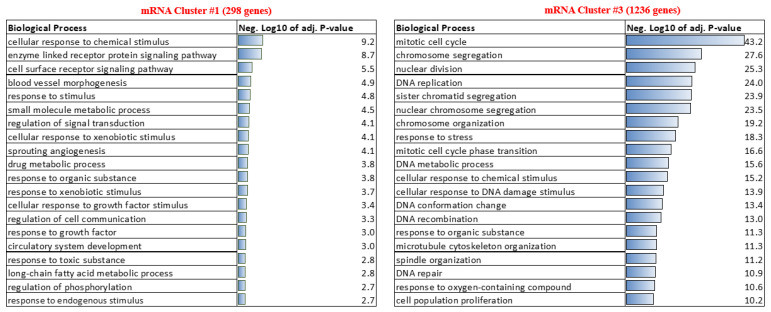
Pathway enrichment analysis of genes identified in lncRNA-mRNA co-expression networks. Two main clusters of mRNA were found to be associated (Pearson’s correlation coefficient, *p*-value < 0.05, −0.8 < R > 0.8) to lncRNA expression. The genes in cluster #1, left panel, were predominantly involved in biological processes related to cellular response to chemical, xenobiotics, or drug stimulus. Cluster #3, with 4 times more genes (right panel), was highly enriched for genes that control the cell cycle, chromosomal organization, and DNA damage/repair response.

To investigate the physiological relevance of ENM-triggered DE lncRNAs, we performed separate functional enrichment analysis of the mRNAs in the top two co-regulated mRNA-lncRNA clusters depicted in Figure 3A. Cluster #1 consisting of 298 unique mRNA transcripts and Cluster #3 consisting of 1236 unique mRNA transcripts were subjected to Gene Ontology based biological process enrichment analysis, using the mouse genome (Figure 4). The genes from cluster #1 were notably found to be involved in cellular response to chemical stimulus (adj. *p*-value 7.07 × 10^−10^), cellular response to growth factor stimulus (adj. *p*-value 1.56 × 10^−3^), and response to toxic substance (adj. *p*-value 4.37 × 10^−4^). The most notable biological processes enriched by genes in cluster #3 were mitotic cell cycle (adj. *p*-value 6.32 × 10^−44^), chromosome segregation (adj. *p*-value 2.24 × 10^−28^), cellular response to DNA damage stimulus (adj. *p*-value 1.20 × 10^−14^), response to oxygen-containing compound (adj. *p*-value 2.75 × 10^−11^), and response to cytokine (adj. *p*-value 5.10 × 10^−8^). Due to its superior gene set size, it is not surprising that almost 5 times biological processes are enriched by the genes in cluster #3 (372 biological process with adj. *p*-value < 0.05) when compared to the genes in cluster #1 (78 biological process with adj. *p*-value < 0.05). However, it is interesting to note that about 95 of the top 100 biological processes enriched by the genes in cluster #3 are described in terms of chromosomal organization, DNA conformation, or DNA damage. A list of the lncRNA-correlated mRNAs from cluster #1 and #3 is provided as Supplementary File #1. The top 20 biological processes represented by the genes in each cluster are provided in Figure 4. Because majority of the lncRNA-associated genes were identified in cluster #3 (1236 genes), ENM-specific pathway enrichment analysis was carried out for the genes in this cluster. Venn comparisons and a list of the top enriched pathway for each ENM class are shown in Figure S4. As expected, the most significantly enriched pathways (cell cycle regulation, DNA damage response, and chromosomal organization) were common to each of the CuO, MWCNT, and TiO_2_p exposures. Unique pathways were less significant, but still related to DNA damage repair or cell cycle regulation (CuO and MWCNT). Unique pathways triggered by lncRNA-associated genes in TiO_2_p exposures corresponded to immune cell (granulocytes and mononuclear cell) migration, and regulation of cytokine production.

### 3.5. The Relative Expression of LncRNAs Predicted to Be Predominantly Involved in Regulation of Cellular DNA Damage Response, Ranks ENM from Least to Most Toxic

**Figure 5:**
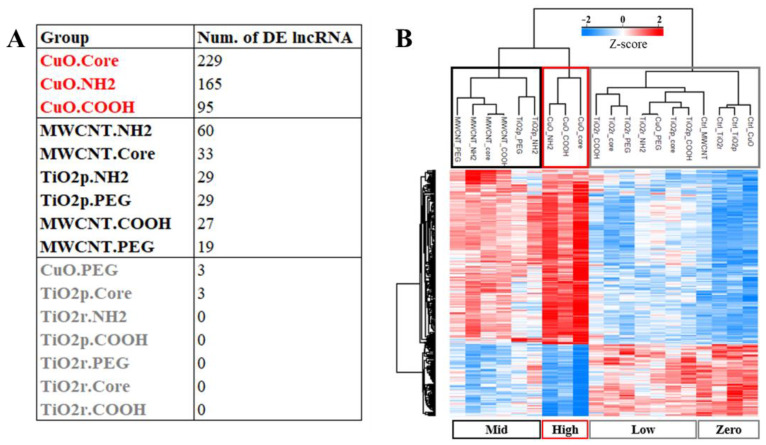
Categorization of ENM toxicity based on expression of lncRNAs involved in regulation of DNA damage and repair response. In (**A**), the frequency distribution of DE lncRNA transcripts across the various engineered nanomaterial (ENM) exposures is shown. The highest number of these regulatory lncRNAs were identified as differentially expressed in CuO exposures, with the exception of CuO-PEG (red font), followed by all MWCNT plus TiO_2_p-NH_2_/PEG (black font). A few to none of these lncRNAs were differentially expressed in all TiO_2_r exposures, CuO-PEG, and TiO_2_p-Core/COOH (gray font). A heatmap of the hierarchical clustering that is based on the average relative (Z-score) expression of these lncRNAs across all sample groups is shown in (**B**). Clusters of the sample groups that ranks the relative toxicity of the ENM in very much the same way as seen in (**A**). That is, from zero (unexposed)/low (gray box) to mid toxicity (black box) and then high toxicity (red box) ENM.

LncRNAs are found to be strongly correlated to two different mRNA clusters (Figure 3, above). One of the mRNA clusters (cluster #3, with 1236 genes) was highly enriched for pathways related to DNA damage and repair processes (Figure 4). We next sought to answer whether the expression profile of the lncRNAs that potentially regulate these mRNA genes, is consistent with the relative toxicity of the ENM investigated. To do this, we filtered out all lncRNAs that were not highly correlated (−0.8 < R > 0.8) to at least 15 of the 1236 genes in mRNA cluster #3. Only 242 of the 817 DE lncRNAs were retained. These 242 lncRNAs are provided in Table S1. The distribution of these DE 242 lncRNAs across the different ENM exposures is shown in Figure 5A. Hierarchical clustering based on the average Z-score normalized log2 intensities of the 242 lncRNA transcripts across all exposure groups is shown as a heatmap in Figure 5B. Several ENM clusters can be seen, the top 3 of which are; one cluster consisting of all control exposures, plus the CuO-PEG, TiO_2_p-Core, TiO_2_p-COOH, TiO_2_r-Core, TiO_2_r-COOH, TiO_2_r-NH_2_, and TiO_2_r-PEG exposures. Another cluster consisted of TiO_2_p-NH_2_ and TiO_2_p-PEG, MWCNT-Core, MWCNT-COOH, MWCNT-NH_2_, and MWCNT-PEG exposures. In the third cluster, we could find the remaining three CuO exposures—CuO-Core, CuO-COOH, and CuO-NH_2_. All three clusters sorted, by the number of significantly DE lncRNAs (exposed/control), and their relative expression across all sample groups are shown in Figure 5A,B, with gray denoting controls (zero) and lower toxicity ENM, black for medium toxicity ENM, and red for higher toxicity ENM. Irrespective of their surface chemistry, all TiO_2_r and MWCNT ENM rank within the lower and medium toxicity clusters, respectively. On the other hand, the effect of PEGylation is more obvious for CuO ENM, where CuO-PEG clusters together with unexposed and lower toxicity ENM, while CuO-Core, -COOH, and -NH_2_ are the nanoparticles with the highest relative toxicity amongst these panel of engineered nanomaterials.

## 4. Discussion

We have recently published [6] that CuO and MWCNT were the most toxic materials among the 28 tested ENM in terms of strong neutrophilic and eosinophilic cell influx, nuclear dust formation, and mucus hypersecretion in mouse lungs. Additionally, the spherical and rod-shaped TiO_2_ nanomaterials induced comparable macrophage influx with mild biological effects. These materials also caused perturbations in cell division, innate immune response, and inflammatory response pathways. Epigenetic disturbance via modulation of lncRNA expression, has increasingly been observed to play key roles in the toxicity of environmental chemicals like benzene, cadmium, lead, and nickel [22]. In particular, siRNA-mediated knockdown of overexpressed lncRNAs highlighted their role as key mediators of the DNA damage response triggered by exposure to cadmium [23]. To identify the role of lncRNA in the biological responses of different ENM with different surface chemistries, we have investigated expression patterns in protein-coding and non-coding genes from the same samples. We profiled material-specific changes in lncRNA expression and provided insight into their potential roles by constructing coding-non-coding gene co-expression networks, based on Pearson’s correlation coefficients of no less than 0.80.

At the level of the core material, CuO triggered the highest number of differentially expressed transcripts (DET). This was also true for all surface modifications, except with the PEG functional group, where PEGylation suppressed the toxicity of CuO. With a 6-fold decrease in the number of DET, PEGylation of CuO was the most effective at reducing the toxicity of the core ENM. Previous measurements of zeta potential revealed that PEGylation decreased the net electrical charge of CuO from +14 to −17 [6]. This is consistent with findings that polyethylene glycosylation (PEG), generates a hydrophilic surface that shields the core particle from immunosurveillance [5]. In fact, with these same particles, we showed that CuO triggers increased infiltration of neutrophils and lymphocytes, which are both diminished by PEGylation [5,6]. This absence of immune cell infiltrates may explain why relatively few DET were observed in mice exposed to CuO-PEG, when compared to control mice.

The functionalized ENM formed three different clusters based on their differentially expressed lncRNA transcripts. TiO2p-PEG and TiO2p-NH2 formed a distinct cluster, although all other functionalizations of TiO2p/s clustered with the negative controls. MWCNT and CuO groups, except CuO-PEG, formed a third cluster with specific changes in lncRNA expression pattern. TiO2 ENM have shown minimal ability to induce gene expression in our studies [24,25,26], nearly all mutagenicity tests are reported negative [27], and they are often considered as control particles in animal models. However, growing number of evidence suggests that instead of direct biological effects, TiO2 ENM might cause harm after prolonged exposure and are suggested to play a role in the development of inflammatory bowel disease (IBD) and colon cancer after ingestion [28]. Therefore, their use as a food additive has been banned since the beginning of 2020 in France (USDA Foreign Agricultural Service, GAIN Report Number: FR1917). A recent study suggests that cellular uptake of TiO2 ENM induces dose-dependent oxidative stress and alterations in microRNA expression [29]. Our results support the idea that TiO2p ENM are not toxicologically inert and do induce changes in epigenetic regulation, via shared as well as unique mechanisms when compared to the other tested ENM. In fact, unique biological processes enriched by lncRNA-associated DEG were related to immune cell (mononuclear cells, granulocytes, and eosinophils) migration.

A lncRNA-gene co-expression network analyses based on Pearson’s correlation coefficient, R > 0.8 to at least 15 genes, identified a set of 242 lncRNAs, whose relative expression profiles separate the exposed mice into 4 categories of unexposed, low, medium, and high relative toxicities. This ranking is consistent with in vitro cytotoxicity of the same materials in human primary macrophages and a THP-1 cell line [15]. These 242 lncRNAs were upregulated relative to control exposures and were highly associated with genes involved in regulation of cell cycle, chromosomal organization, and DNA damage response (Figure 4, right panel), suggesting these are shared mechanisms that explain the relative cytotoxicity of these set of ENM. At the selected cut-offs for significant differential expression, increased expression of the majority (198 transcripts) of the lncRNAs involved in mRNA-mediated regulation of chromosomal organization and DNA damage response was triggered by exposure to CuO nanoparticles, followed by MWCNT (51 transcripts) and TiO_2_p (42 transcripts) ENM. These lncRNAs were positively correlated to a cluster of 1236 genes–of which most (1144) were differentially expressed in response to CuO exposure, 581 in response to MWCNT, and 326 in response to TiO_2_p exposures. The most significant pathways enriched by lncRNA-associated genes correspond to cell division and associated functions including DNA replication, chromosome organization, response to stress, and DNA damage response. This finding is consistent with previous reports that a large repertoire of lncRNAs is able to interact with chromatin-modifying complexes [11] and supports our approach of using mRNA correlation analysis for functional prediction of differentially expressed lncRNAs. In addition, some of the biological processes represented by lncRNA-associated genes were consistent with previous in vivo and in vitro studies investigating adverse exposures to similar engineered nanomaterials. For example, CuO exposure is known to induce wide stress responses, inflammation and direct inhibition of transcription [30], while MWCNT cause, in addition to eosinophilic inflammation, oxidative stress-dependent lung fibrosis and goblet cell metaplasia [31,32]. For the less toxic TiO_2_p, these lncRNAs appear to have a more unique role, which may reflect long(er)-term adverse biological effects of TiO_2_p that will otherwise have little to no acute toxicity.

## 5. Conclusions

In conclusion, the immunomodulatory effects of CuO and MWCNT appear to be primarily mediated via lncRNA-independent mechanisms, whereas in TiO_2_p exposures, a subset of lncRNA-regulated DEG that are involved in immune cell migration were identified. In all exposures, mitotic cell division as well as cellular response to DNA damage and repair were the most significant lncRNA-dependent biological processes affected by ENM exposure. The fact that the expression of lncRNA transcripts from lncRNA-mRNA co-regulated networks of DNA damage and repair response pathways, clusters the different ENM according to their cytotoxic potential, emphasizes the biological relevance of lncRNA expression profiling in toxicogenomic studies. There is also a case to be made for investigating changes in DNA-associated lncRNA expression as proxies for ENM genotoxicity. The data show that exposure to ENM induces different sets of significantly altered non-coding RNAs, whose expression is associated to changes of mRNA expression in mouse lungs. LncRNAs identified in this study and mRNA genes identified in our previous study complement each other, and their co-expression profiles provide tools to be used as biomarkers for classification and estimation of toxicity of different ENM. In addition, given that the lncRNA expression profile (number of differentially expressed transcripts and their association with genes involved in chromatin organization) flagged the supposedly inert TiO_2_p-PEG and TiO_2_-NH_2_ particles as significantly toxic ENM, we propose that when possible lncRNA expression be integrated in the development of adverse outcome pathways—especially for particles that may not trigger drastic acute changes in the expression of protein-coding genes. This study deepens our understanding on the interaction between mRNA and lncRNA and broadens the avenues to evaluate particle toxicity, which is essential for development of more robust predictive models to speed up toxicity assessment of ENM.

## Data Availability

The data presented in this study are openly available via gene expression omnibus, accession number GSE157266.

## Supplementary Materials

The following are available online at https://www.mdpi.com/article/10.3390/cells10051085/s1, Figure S1: Principal component analysis based on differentially expressed long non-coding RNA (no fold change cutoff); Figure S2: Comparison of ENM-induced mRNA and lncRNA expression in total RNA isolated from mice lung tissue samples; Figure S3: Relative median lncRNA expression intensities of transcripts that are highly correlated (abs Pearson correlation coefficient, R > 0.8) to DE mRNA from the same ENM exposures. Co-regulated lncRNA-mRNA networks, triggered by hazardous copper oxide (CuO), multiwalled carbon nanotubes (MWCNT) and titanium dioxide (TiO_2_p/TiO_2_r) engineered nanomaterial exposures comparison of ENM-induced mRNA and lncRNA expression in total RNA isolated from mice lung tissue samples. Relative to controls, correlated lncRNA transcripts were predominantly upregulated (ncRNA #1) or downregulated (ncRNA #3). Data bars are mean plus standard deviation of the normalized transcript expression intensity from 3 biological replicates; Figure S4: Number of biological processes enriched by lncRNA-associated genes. Particle-specific pathway enrichment analysis of lncRNA-associated differentially expressed genes (DEG) from mRNA cluster #3 (1236 genes). The top shared pathways of toxicity (black box) correspond to cell cycle, DNA damage, oxidative stress (cellular response to oxygen-containing compound), and inflammatory response. Biological processes listed in the purple, green, and yellow boxes represent the most significant pathways that are uniquely enriched by lncRNA-associated DEG in CuO, TiO_2_p, and MWCNT exposures, respectively; Table S1: 242 lncRNAs.
