# Supplementary material for: Profiling Non-Coding RNA Changes Associated with 16 Different Engineered Nanomaterials in a Mouse Airway Exposure Model
Source: Cells. 2021 May 1;10(5):1085. doi: 10.3390/cells10051085 (PMC8147388; doi:10.3390/cells10051085)
Supplement: Supplementary file 1 [file cells-10-01085-s001.zip › Supplementary Figures S1 - S4.pdf]

Supplementary Figures, S1, Figure S2, Figure S3, and Figure S4. Supplementary Table S1

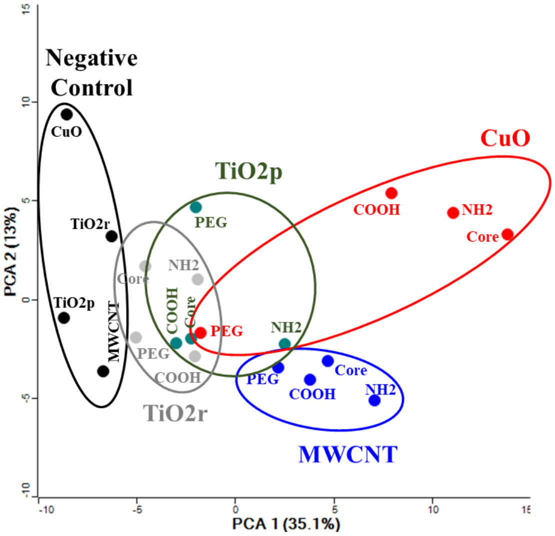

Figure S1. Principal component analysis based on differentially expressed long non-coding RNA (no fold change cutoff). The top two principal components, explaining 48% of the variation between these non-coding transcripts, separates unexposed mouse lung (negative control) from mouse lungs exposed to either rod-like titanium dioxide (TiO2r), spherical titanium dioxide particles (TiO2p), multi-walled carbon nanotubes (MWCNT) and copper oxide (CuO) nanomaterials. Core, COOH, NH2, and PEG, represent pristine, carboxylated, aminated, and pegylated material surface chemistries, respectively.

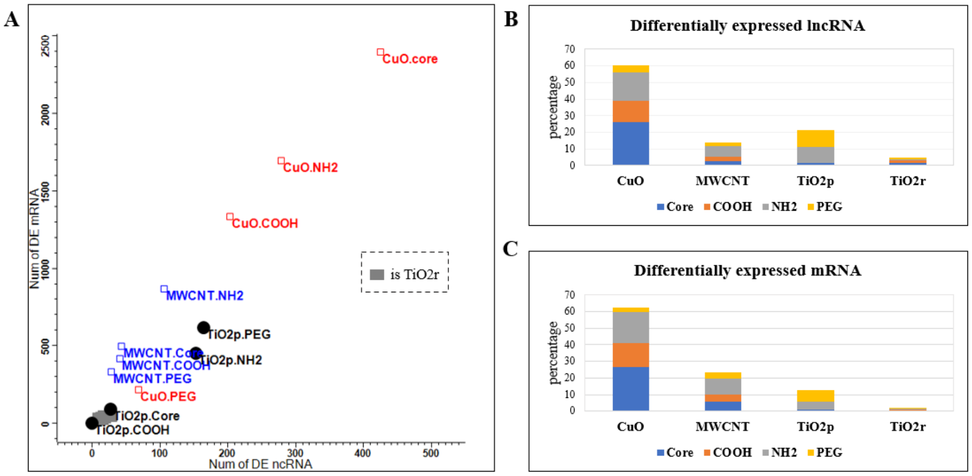

Figure S2. Comparison of ENM-induced mRNA and lncRNA expression in total RNA isolated from mice lung tissue samples. In terms of the number of differentially expressed transcripts, both layers of the transcriptome show very good exposure severity concordance (Spearman's rank correlation of 0.96) (**A**). The number of DE transcripts for each class of ENM particles, expressed as a ration of the total number of differentially expressed transcripts, is shown for lncRNAs (**B**) and mRNA (**C**). Within the lncRNAs, CuO, MWCNT, TiO2p, and TiO2r accounted for 60%, 14%, 21%, and 5% of all DE transcripts, respectively. Meanwhile within the mRNA layer of the transcriptome, 62%, 23%, 13%, and 2% of all DE transcripts was explained by exposure to either CuO, MWCNT, TiO2p, or TiO2r ENM, respectively. For both layers of the transcriptome, a 1.5-fold change and maximum FDR of 5% was implemented to consider a transcript as significantly differentially expressed.

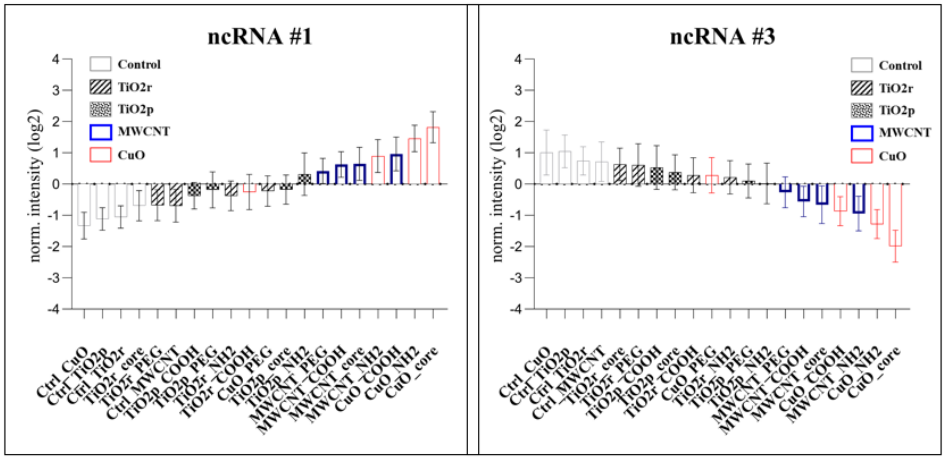

Figure S3. Relative median lncRNA expression intensities of transcripts that are highly correlated (abs Pearson correlation coefficient, R > 0.8) to DE mRNA from the same ENM exposures. Co-regulated lncRNA-mRNA networks, triggered by hazardous copper oxide (CuO), multiwalled carbon nanotubes (MWCNT) and titanium dioxide (TiO2p/TiO2r) engineered nanomaterial exposures comparison of ENM-induced mRNA and lncRNA expression in total RNA isolated from mice lung tissue samples. Relative to controls, correlated lncRNA transcripts were predominantly upregulated (ncRNA #1) or downregulated (ncRNA #3). Data bars are mean plus standard deviation of the normalized transcript expression intensity from 3 biological replicates.

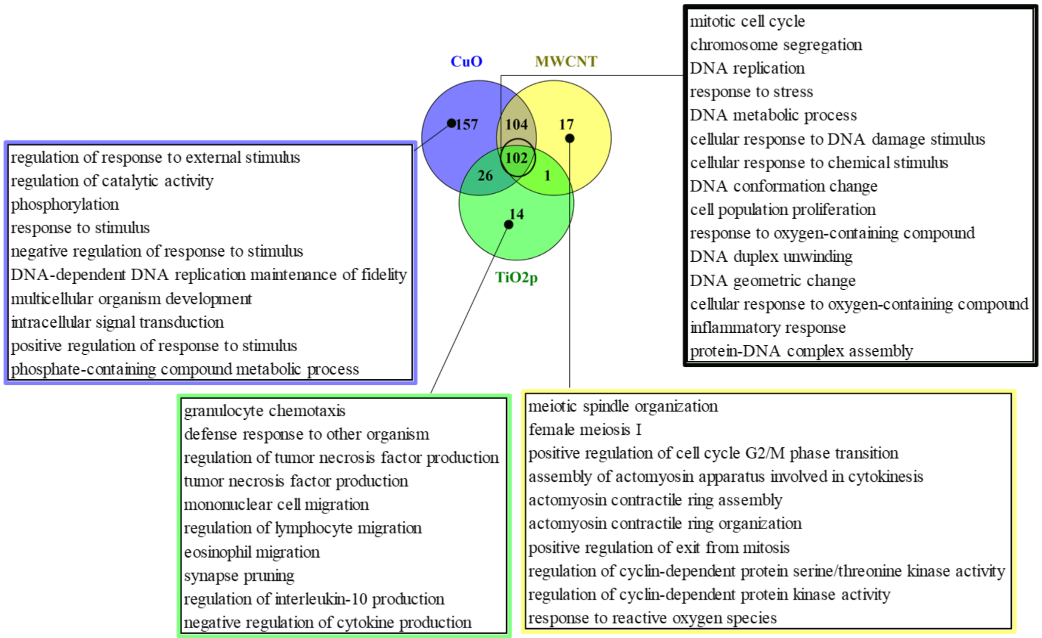

Figure S4. Number of biological processes enriched by lncRNA-associated genes. Particle-specific pathway enrichment analysis of lncRNA-associated differentially expressed genes (DEG) from mRNA cluster #3 (1236 genes). The top shared pathways of toxicity (black box) correspond to cell cycle, DNA damage, oxidative stress (cellular response to oxygen-containing compound), and inflammatory response. Biological processes listed in the purple, green, and yellow boxes represent the most significant pathways that are uniquely enriched by lncRNA-associated DEG in CuO, TiO2p, and MWCNT exposures, respectively.
